# Recent Advances of Representative Optical Biosensors for Rapid and Sensitive Diagnostics of SARS-CoV-2

**DOI:** 10.3390/bios12100862

**Published:** 2022-10-12

**Authors:** Meimei Xu, Yanyan Li, Chenglong Lin, Yusi Peng, Shuai Zhao, Xiao Yang, Yong Yang

**Affiliations:** 1 State Key Laboratory of High-Performance Ceramics and Superfine Microstructures, Shanghai Institute of Ceramics, Chinese Academy of Sciences, 1295 Dingxi Road, Shanghai 200050, China; 2 Graduate School of the Chinese Academy of Sciences, No.19(A) Yuquan Road, Beijing 100049, China; 3 Center of Materials Science and Optoelectronics Engineering, University of Chinese Academy of Sciences, Beijing 100049, China

**Keywords:** optical biosensors, SARS-CoV-2 detection, point-of-care diagnostics

## Abstract

The outbreak of Corona Virus Disease 2019 (COVID-19) has again emphasized the significance of developing rapid and highly sensitive testing tools for quickly identifying infected patients. Although the current reverse transcription polymerase chain reaction (RT-PCR) diagnostic techniques can satisfy the required sensitivity and specificity, the inherent disadvantages with time-consuming, sophisticated equipment and professional operators limit its application scopes. Compared with traditional detection techniques, optical biosensors based on nanomaterials/nanostructures have received much interest in the detection of SARS-CoV-2 due to the high sensitivity, high accuracy, and fast response. In this review, the research progress on optical biosensors in SARS-CoV-2 diagnosis, including fluorescence biosensors, colorimetric biosensors, Surface Enhancement Raman Scattering (SERS) biosensors, and Surface Plasmon Resonance (SPR) biosensors, was comprehensively summarized. Further, promising strategies to improve optical biosensors are also explained. Optical biosensors can not only realize the rapid detection of SARS-CoV-2 but also be applied to judge the infectiousness of the virus and guide the choice of SARS-CoV-2 vaccines, showing enormous potential to become point-of-care detection tools for the timely control of the pandemic.

## 1. Introduction

In December 2019, an unprecedented pneumonia case was first reported in Wuhan, a city in China, and quickly spread around the world [1]. The Coronavirus Study Group (CSG) of the International Committee on Taxonomy of Viruses (ICTV) officially named the novel coronavirus as severe acute respiratory syndrome coronavirus 2 (SARS-CoV-2) on 11 February 2020 [2]. The surface of SARS-CoV-2 is mainly composed of four structure proteins, including spike glycoprotein (S), which specifically binds to host cell receptors, membrane protein (M), which is related to envelope formation, envelope small membrane protein (E), and nucleoprotein, which is related to viral assembly (N). SARS-CoV-2 enters body tissues rapidly and initiates infection when the S protein binds to the angiotensin-converting enzyme 2 (ACE2) on the surface of the host cell [3,4]. To date, more than 0.6 billion infections have been reported around the world, and the surprisingly fast propagation is responsible for the pandemic. The virus can be transmitted primarily through three routes: (1) respiratory droplets and close contact; (2) contact with virus-contaminated items; and (3) exposure to high concentrations of aerosols in a relatively closed environment. According to reports, SARS-CoV-2 can not only damage various organs, including the lung, heart, kidney, and brain, but can also cause serious health and mental problems after recovery [5,6,7]. Particularly, the recent outbreaks of novel coronavirus pneumonia (COVID-19) in various countries and the emergence of variants have increased infectivity, posing an enormous threat to people’s lives and health, and have brought immeasurable pressure to the global medical system. Therefore, the early diagnosis of infection and cutting off the transmission route are the keys to effectively controlling the epidemic. At the same time, the development of rapid, sensitive, convenient, and accurate virus detection technology is imminent [8,9,10].

Current clinical diagnostic methods for COVID-19 mainly include molecular (ribonucleic acid (RNA)), antigen, antibody, or imaging tests [2,11]. The reverse transcription polymerase chain reaction (RT-PCR) for the detection of the RNA of SARS-CoV-2 is regarded as the gold standard in epidemic prevention and control because of its high sensitivity and specificity. However, professional operators and equipment are required, and the entire process takes more than 2 h. Furthermore, false negative signals are prone to occur due to varying viral loads during different infection periods [12,13]. Enzyme-Linked Immunosorbent Assay (ELISA) is another commonly used immunological experimental technology and offers the merits of semi-quantitative or qualitative analysis skills based on the color depth but still needs complicated operations, and the accuracy can be easily affected by interference [3]. Likewise, lateral flow immunochromatography (LFIA) based on colloidal gold color reaction has attracted much attention owing to the simple operation, low cost, and strong specificity [14]. As a paradigm, Ge et al. developed the “COVID-19 IgM Antibody Detection Kit”, which has become one of the first batches of rapid detection reagents that have passed the verification of statutory testing institutions. The test kit shows a specificity of 99.6%, as well as an accuracy of 98.1%, and it is considered to be a point-of-care (POC) diagnostics technology that can be used for large-scale screening [15]. Nonetheless, the LFIA is poor in terms of quantitative analysis ability and sensitivity, as it only relies on visual detection.

In light of the tremendous pressure of COVID-19 prevention and control, scientists have been working hard to explore more convenient, rapid, and accurate POC diagnosis technologies. It is worth pointing out that the emergence of nanobiosensors has greatly promoted the development of POC technology. The nanobiosensors are mainly composed of three parts, namely, the identification element (analyte to be measured), the sensitive unit, and the transducer that can convert the biological response into a visual physical or chemical signal. According to the type of output signal, it can be divided into electrochemical biosensors, optical biosensors, etc. [16,17,18]. Among them, optical biosensors exhibit broad application prospects in the field of POC diagnosis in virtue of their high sensitivity, low cost, simple operation, and rapid analysis abilities [19,20]. Recently, a few teams have summarized and prospected the technologies for detecting SARS-CoV-2. For example, Naikoo et al. reviewed the current strategies for the diagnosis of SARS-CoV-2 [21]. The review introduces the merits and shortcomings of traditional detection techniques such as PCR and ELISA in detail and also discusses the research progress of current biosensors including surface plasmon resonance biosensors, multifunctional gene sensors, and electrochemical sensors, which have certain guiding significance for researchers. Thapa et al. discussed smart biosensors for SARS-CoV-2 diagnosis according to the types of nanomaterials, including metal and metal oxide-based nanobiosensors, carbon-based nanobiosensors, and polymer-based biosensors [22]. It provides a foundation for the development of new materials for nanobiosensors. However, there is no systematic discussion of optical sensors in the review. Although Lukose et al. described the development and prospects of optical technologies for virus detection, such as Raman spectroscopy, Fourier transforms infrared spectroscopy, and fluorescence technology for virus detection, which can help people understand the current development of optical sensors to a certain extent, the discussion of fluorescent sensors in the paper mainly focuses on their applications in the detection of HPV, HIV, and HBV viruses [23]. To the best of our knowledge, at present, the review of optical sensors—particularly, fluorescence, Raman, Surface Plasmon Resonance (SPR), and additional biosensors—in the detection of SARS-CoV-2 is relatively rare.

**Scheme 1:**
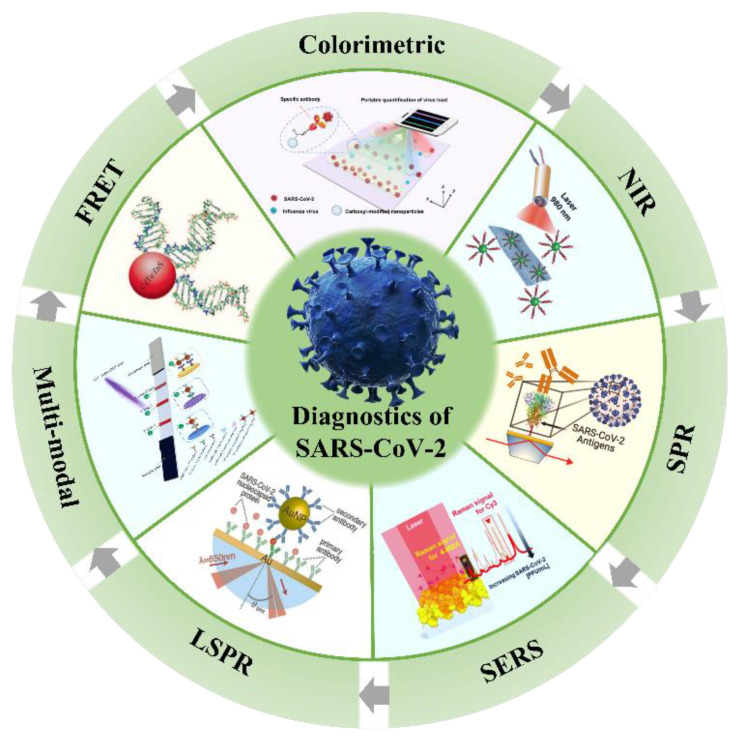
Schematic illustration of optical biosensors for SARS-CoV-2 detection.

Herein, we provide a review on the application of advanced optical sensors in COVID-19 detection. It mainly concentrates on the research progress of novel near-infrared fluorescence biosensors, fluorescence resonance energy transfer biosensors, dual-mode optical biosensors, and surface-enhanced Raman scattering biosensors, with examples in SARS-CoV-2 diagnosis, as shown in Scheme 1. The promising strategies to improve optical biosensors are also discussed.

## 2. Potential Optical Biosensors

The optical sensors mainly analyze the optical signals (absorption, polarization, intensity, wavelength, or refractive index) generated during the combination of the target and the identification element and directly convert or amplify them via the transducer in real time to achieve a qualitative and quantitative analysis of the target. The characteristics of high sensitivity, good selectivity, simple operation, integration, and fast response make them widely used in environmental detection, biotechnology, food safety, medical diagnosis, and so on [24]. In the past decade, optical biosensors have developed rapidly, and many optical biosensing platforms have been used for the detection of nucleic acids, proteins, and exosomes, including but not limited to colorimetric biosensors, fluorescence biosensors, surface-enhanced Raman scattering biosensors (SERS), and surface plasmon resonance biosensors (SPR or LSPR). Notably, the optical sensors do not require complex nucleic acid amplification steps and post-processing in virus detection, which is considered a potential POC diagnostic tool and is expected to play a role in the large-scale screening of COVID-19, especially in low and middle-income regions and countries [25].

**Figure 1:**
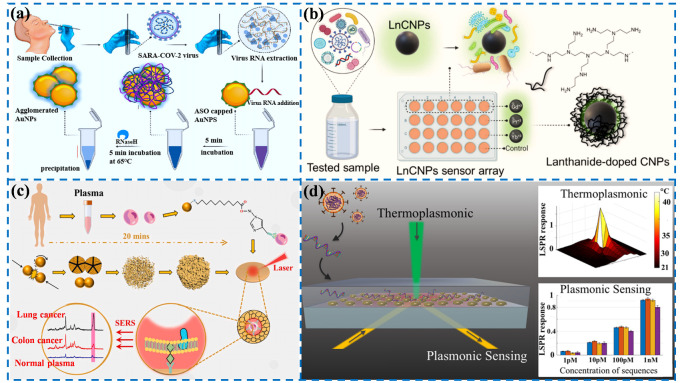
Schematic diagram of optical sensors in biological detection: (**a**) colorimetric sensor for the detection of RNA from SARS-CoV-2. Reprinted with permission from ref. [26]. (**b**) Fluorescent sensor for the identification of SARS-CoV-2 in wastewater. Reprinted with permission from ref. [27]. Copyright 2022, American Chemical Society. (**c**) SERS sensor for the detection of tumor cell exosomes. Reprinted with permission from ref. [28]. Copyright 2022, Elsevier. (**d**) Plasma sensor for the detection of SARS-CoV-2. Reprinted with permission from ref. [29]. Copyright 2020, American Chemical Society.

Colorimetric sensing technology can perform colorimetric analysis of the target through the visible color change of the solution, without the help of sophisticated instruments. The method is a semi-quantitative analysis and is pretty simple and convenient but has a low accuracy and can only be measured in a limited range. Moitra’s team designed a colorimetric assay based on AuNPs that can diagnose isolated RNA samples within 10 min [26]. The thiol-modified antisense oligonucleotides of SARS-CoV-2 were modified on the surface of Au NPs. The single Au NPs will aggregate when the RNA of SARS-CoV-2 is present in the detection sample, and the precipitate is observable after using ribonuclease to cut the RNA chain in the solution. The limit of detection (LOD) of the sensing assay reaches 0.18 ng/μL, providing a reliable method for disease diagnosis (Figure 1a).

Fluorescence sensing mainly conducts a qualitative and quantitative analysis of the target by determining the excitation and emission wavelengths and detecting the changes of the fluorescence intensity, which is commonly accompanied by the enhancement, quenching, or change in the emission signal. As we all know, the presence of asymptomatic infections has brought enormous pressure to epidemic prevention and control. At present, relevant studies have shown that the detection of viruses in community sewage, namely, wastewater-based epidemiology (WBE), may be an effective strategy for the rapid diagnosis of the COVID-19 epidemic. Alafeef et al. reported a novel fluorescent sensing array based on three different lanthanum-doped carbon nanoparticles (LnCNPs), as shown in Figure 1b [27]. The LnCNPs were prepared by a hydrothermal process. The SARS-CoV-2 can be distinguished from other viruses or bacteria through machine learning according to differential fluorescence response patterns due to the counterion–ligand interactions. The LOD of the sensing array was 1.5 copies/μL, showing great promise in preventing the spread of the epidemic.

SERS refers to the phenomenon of an enhanced Raman signal generated on the surface of some rough nanomaterials. The technology is provided with high sensitivity, and the detection ability of some SERS-active substrates can even reach the single molecule level. Therefore, the active substrates are essential to achieving excellent SERS performance. The properties of nanomaterials, such as high surface energy, agglomeration and dispersion states, surface plasmon resonance, etc., can affect the activity of SERS substrates. Our group reported a novel nano-porous gold SERS chip functionality with CP05 polypeptide, which can specifically capture exosomes, as depicted in Figure 1c [28]. The lung and colon cell exosomes can be distinguished from normal plasma at the single vesicle level by combining Raman spectroscopy and machine learning methods without any labels or the purification of exosomes, providing a new idea for studying exosomes at the spectral level.

The principle of surface plasmon resonance (SPR) biosensors is to detect the target substances through the change in the refractive index caused by the interaction between the plasma resonance wave and the target molecules on the metal surface. The bifunctional plasmonic system developed by Qiu’s team possesses highly sensitive, rapid, and reliable diagnostic potential for the detection of SARS-CoV-2 [29]. The dual-function biosensor integrates the PPT effect and LSPR-sensing transduction onto a cost-effective gold nanoislands (AuNIs) chip. The PPT and LSPR effects can be excited at different wavelengths by using two different angles of incidence, significantly enhancing sensing stability, sensitivity, and reliability (Figure 1d). More importantly, the in situ PPT photothermal enhancement on the AuNI chip significantly improves the hybridization kinetics and nucleic acid detection specificity. The elaborated dual-function LSPR biosensor could provide a reliable and easily implementable diagnostic platform to improve the diagnostic accuracy of clinical tests.

The rapid development of nano-biotechnology has opened up infinite possibilities for the development of new detection technologies, especially the emergence of nanofluorescent materials, providing a wide range of prospects for the application of novel fluorescence detection techniques. As mentioned above, fluorescence sensors, colorimetric sensors, SERS sensors, and SPR sensors exhibit fast and high-sensitivity properties in biological detection, which are of great significance for realizing the timely detection of SARS-CoV-2. Next, we will discuss the characteristics and application progress of these sensors in detail.

## 3. Fluorescence Biosensor for SARS-CoV-2 Diagnosis

Fluorescence biosensors are a common method for the on-site detection of infectious diseases, which possess the advantages of high sensitivity, low cost, and simple operation [30,31]. Fluorescent molecules are the most important component in the construction of fluorescent biosensors, including fluorescent dyes and fluorescent nanomaterials. The Fluorescence Resonance Energy Transfer (FRET) mechanism is often introduced in designing fluorescence biosensors, involving the energy transfer from a fluorescent donor to a fluorescent acceptor. Additionally, some nanomaterials can generate fluorescence on their own, which is widely used in fluorescent biosensors due to their unique physical, chemical, and electron transport properties [32].

### 3.1. FRET Biosensors for SARS-CoV-2 Detection

Fluorescence Resonance Energy Transfer (FRET) was first proposed by Föster in 1948, so it is also called Föster energy transfer, which is a typical non-radiative energy transfer process [33]. FRET probes with multiple fluorescence analysis, high sensitivity, and easy operation have been rapidly developed in the field of biosensing and have become a powerful tool for the detection of the target molecule in the biosensing field [34,35,36]. The preconditions of FRET are that the emission spectrum of one fluorescent molecule (also known as the donor molecule) overlaps with the excitation light of the other fluorescent molecule (also known as the acceptor molecule), and the distance between them is less than 10 nm. Then, the excitation energy of the donor is used to induce fluorescence emission from the acceptor, while the fluorescence intensity of the donor is reduced. In particular, the selection of the donor and acceptor is crucial for achieving an efficient FRET process.

**Figure 2:**
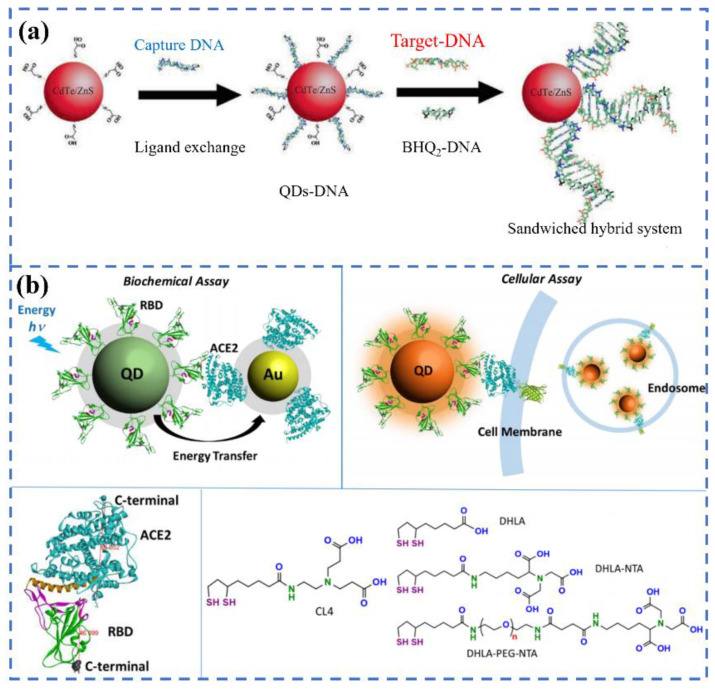
The schematics of the DNA-conjugated CdTe/ZnS QDs nanoprobe for the detection of the target DNA derived from the COVID-19 virus genome(**a**). Reprinted with permission from ref. [38]. Copyright 2022, Elsevier. The energy transfer process from QD-RBD to AuNP-ACE2 and the corresponding cellular assay (**b**). Reprinted with permission from ref. [39].

Among the candidate materials, quantum dots (QDs) with the features of good dispersion, high quantum yield, stable optical activity, and excellent photoluminescence performance have garnered much interest [37]. For example, Bardajee et al. designed CdTe-ZnS QDs functioned with DNA (QDs-DNA) to specifically recognize the DNA or RNA of the COVID-19 virus via the FRET method [38]. The QDs-DNA acts as a donor molecule, and the BHQ_2_-DNA was prepared to act as an acceptor molecule in the FRET process, as can be seen in Figure 2a. When combined with target RNA, the fluorescence of QDs-DNA can be extremely quenched by BHQ_2_-DNA in 25 min under an excitation of 325 nm, and the limit of detection was evaluated to be 0.000823 μM. The presence of thiolate-captured DNA oligonucleotides on the surface of QDs is responsible for the high sensitivity, because it can facilitate the interactions between QDs and target DNA. In another study, Gorshkov et al. provided a FRET-based biosensor to monitor the interactions of the spike proteins receptor binding domain to the host cell’s ACE2 receptor (Figure 2b). In the system, the QDs were conjugated with the recombinant spike receptor binding domain as a donor (QD-RBD), and ACE2-conjugated gold nanoparticles were regarded as an acceptor (AuNP-ACE2). It was shown that the QD-RBD and ACE2 are tightly bound and can enter the cell via the receptor-mediated endocytosis that is dependent on clathrin. This assay can be used for the high-throughput screening and detection of SARS-CoV-2 virus particles [39]. In addition, organic dyes are often used in the FRET system. Recently, Bardajee et al. established a novel cyanine 3 (Cy3)-based bio-conjugated sensor to rapidly and sensitively identify the specific DNA of COVID -19 via the FRET method. During the detection process, the target DNA, Cy3-DNA probe (donor), and BHQ2-DNA (acceptor) can form a sandwiched hybrid structure. The LOD of the Cy3 probe was calculated to be 0.09945 μM. Although the detection limit is higher than that of QDs-based sensors, it can still be used to analyze real samples [40].

### 3.2. NIR Biosensors for SARS-CoV-2 Detection

Both the excitation and emission peaks of conventional biosensors based on organic dyes, semiconductor quantum dots, etc. locate in the UV-visible region [41]. However, most biomolecules have autofluorescence in the range, which can interfere with the fluorescence signal of the biosensor and reduce the detection sensitivity. Compared with ultraviolet-visible light, biomolecules have ultra-low absorption and scattering in the near-infrared region (700–1700 nm), and the chance of background signal interference is rare. Therefore, the NIR biosensors will enable more accurate and sensitive diagnosis [42,43].

**Figure 3:**
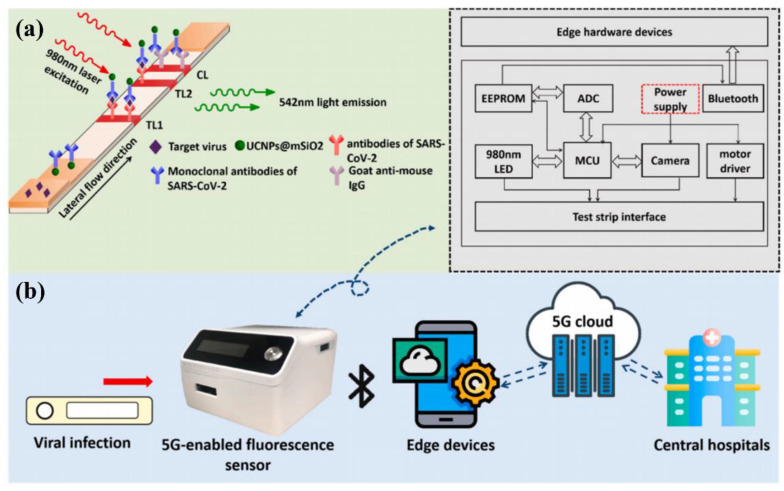
(**a**) The working principle of the detection of S and N proteins from SARS-CoV-2 based on NaYF_4_: Yb, Er@SiO_2_ nanoparticles; (**b**) 5G-enabled NIR-fluorescence sensor used for SARS-CoV-2 diagnosis. Reprinted with permission from ref. [47]. Copyright 2021, Elsevier.

Lanthanide ions (Ln^3+^) possess inherent advantages such as narrow emission peaks, long fluorescence lifetimes, low biological toxicity, and the easy regulation of luminescence properties owing to the shielding effect of the outermost *5s* electrons, attracting extensive attention in the field of biological detection [44,45,46]. Guo’s team reported a 5G fluorescent biosensor based on NaYF_4_: Yb, Er@SiO_2_ upconversion luminescence nanoparticles combined with LIFA to quantitatively detect S and N proteins of SARS-CoV-2. The detection platform has a detection limit of 1.6 and 2.2 ng/mL for S and N proteins, respectively. Additionally, the sensor can transmit personal medical data to a computer or smartphone via Bluetooth. Then, the medical centers can master the situation of patients in time and give professional medical advice on the Internet of Medical Things [47], as depicted in Figure 3. The sensing platform is of great significance for the early detection and treatment of patients infected with SARS-CoV-2.

Lanthanide-doped polyethylene (LNP) nanoparticles are also an excellent candidate for NIR biosensing. For instance, Feng et al. constructed a sensitive immunofluorescence analysis method using the Eu fluorescent microsphere that can rapidly detect the IgM and IgG of COVID-19 in human serum or plasma within 10 min, solving the problem of the rapid quantification of serum antibodies [48]. By testing dozens of positive and negative serum or plasma samples, the results showed that the sensitivity and specificity of the biosensor were 98.72% and 100% (IgG) and 98.68%, and 93.10% (IgM), respectively. Chen et al. prepared lanthanide-doped polysterene nanoparticles via the miniemulsion polymerization method to detect anti-SARS-CoV-2 IgG in human serum within 10 min. The validation of clinical samples showed that the nanoplatform can detect anti-SARS-CoV-2 IgG in human serum rapidly and sensitively and can analyze suspicious cases [49]. Compared with NIR-I, the fluorescence emission in NIR-II can also improve the signal to background auto-fluorescence. Hu and his colleagues fabricated NIR-II nanoparticles by encapsulating organic dyes in polyethylene and used them as a sensing platform for the rapid detection of the SARS-CoV-2 antigen. The biosensor possesses a wide analytical range of 0.02–120 ng/mL, and the LOD is 0.01 ng/mL, which cannot be compared with traditional colloidal gold technology [50]. These results suggest that the NIR-II biosensor with enhanced performances is suitable for mass screening.

**Figure 4:**
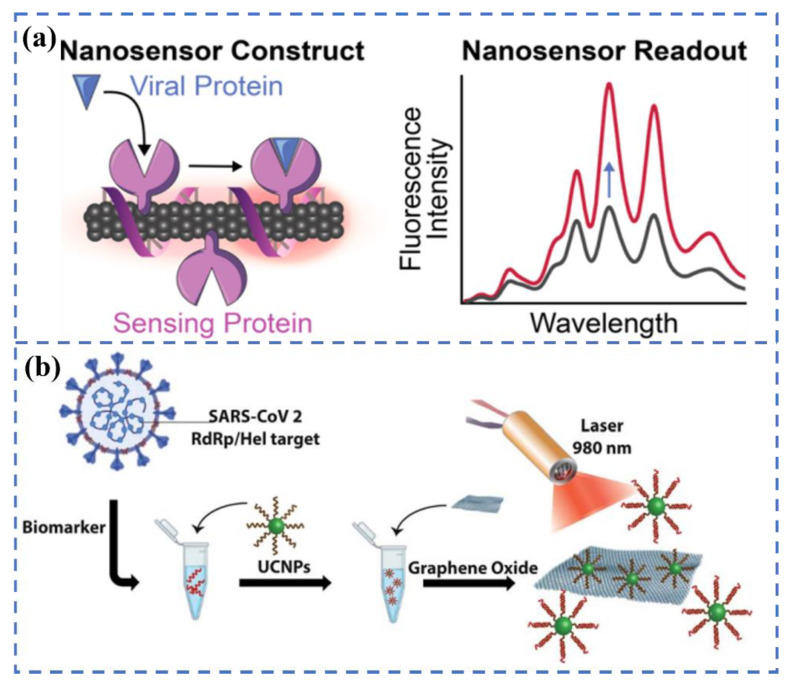
(**a**) Construction of the ACE2-SWCNT nanosensor formation to sense protein ACE2. Reprinted with permission from ref. [52]. Copyright 2021, American Chemical Society. (**b**) Detection of the RdRp/Hel gene of SARS-CoV-2 via an upconversion nanoparticles/graphene-based biosensor. Reprinted with permission from ref. [53].

Carbon materials, as an emerging class of luminescence nanoprobes, have been extensively developed and studied due to their tunable emission, controllable morphology, and good biocompatibility. Several excellent recent articles have highlighted the essential role of carbon-based materials in responding to the COVID-19 pandemic [51]. Single-walled carbon nanotubes (SWCNT) are also considered promising materials for the preparation of fluorescent biosensors due to their intrinsic NIR fluorescence, easy functionalization, and excellent photostability. Pinals et al. developed an optical sensing method based on SWCNT (Figure 4a). The S protein of SARS-CoV-2 can be identified by linking ACE2 to SWCNT through non-covalent bonding [52]. The non-covalent bonding is beneficial to maintaining the inherent luminescent properties of carbon nanotubes. The constructed sensor can rapidly image the S protein in several seconds, with a detection limit of 12.6 nM. In the latest reports, an upconversion nanoparticles/graphene-based biosensor was constructed for the rapid identification of viral oligonucleotide by Alexaki’s team [53]. The upconversion material is functionalized by an oligonucleotide. The oligonucleotide aromatic bases will interact with the graphene oxide (GO) when graphene is present, resulting in the quenching of fluorescence. In the presence of the target virus, the functional upconversion nanoparticles will preferentially bind to the target RNA, thereby reducing fluorescence quenching (Figure 4b). The whole detection process takes 30 min, and the minimum detection limit for this system is 5 fM.

### 3.3. Multi-Modal Biosensors for SARS-CoV-2 Detection

Single-modality detection techniques such as colorimetric, fluorescence, SPR, and SERS techniques—although each of them has its own unique merits—still fail to meet the requirements of practical applications. To overcome the issues that arise in SARS-CoV-2 diagnosis, multimodal detection methods have become a focus for researchers [54,55]. Because the multifunctional biosensors combine the advantages of two or more working units, they can greatly reduce false negative results and improve the accuracy of diagnosis. Additionally, novel biosensors can be used in various scenarios, expanding the application scope. Many groups have also been working on the development of multi-modal biosensors since the outbreak of COVID-19.

**Figure 5:**
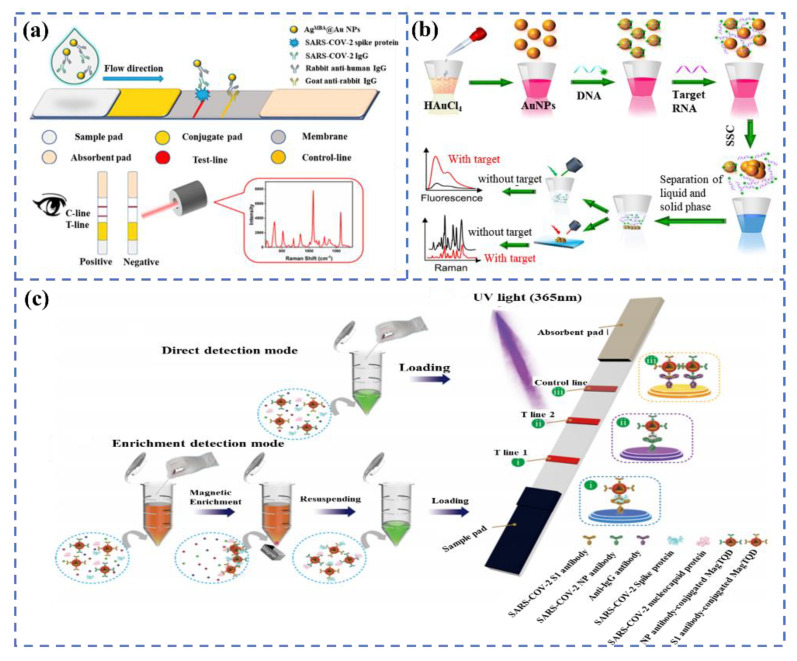
(**a**) The schematic diagram of the colorimetric and SERS dual-mode detection of SARS-CoV-2 based on LFIA. Reprinted with permission from ref. [57]. Copyright 2022, American Chemical Society. (**b**) the preparation process of AuNPs and the tri-mode detection of target RNA. Reprinted with permission from ref. [58]. Copyright 2021, Elsevier. (**c**) Dual-mode LFIA for the simultaneous detection of the S and NP of SARS-CoV-2 by Fe_3_O_4_ nanocomposites with a multilayer QD-Shell. Reprinted with permission from ref. [60]. Copyright 2021, American Chemical Society.

For example, Han et al. proposed a dual-functional LFIA biosensor relying on the SiO_2_@Au/QD nanoparticles to identify the spike 1 (S1) protein of SARS-CoV-2. The performance of the colorimetric and fluorescent was evaluated in real samples, and the detection limits of the detected S1 proteins were 1 ng/mL and 33 pg/mL within 30 min, respectively, indicating that the novel biosensor has brilliant application prospects [56]. As we all know, antibodies and RNA are also significant indexes for diagnosing the SARS-CoV-2 infection. Recently, Liang et al. reported a colorimetric and SERS dual-mode lateral flow immunoassay for the qualitative and quantitative detection of SARS-CoV-2 IgG, with a detection limit of 0.52 pg/mL in 15 min (Figure 5a). The excellent performance is related to the delicate structure of AgMBA@Au, prepared by a ligand-assisted epitaxial growth method [57]. Gao et al. constructed a triple-mode biosensor to detect RNA in SARS-CoV-2 by using the colorimetric, SERS, and fluorescence properties of Au nanoparticles [58]. The system realizes a limit detection of the femtomole level in all modes, which is 160 fM in the colorimetric mode, 259 fM in the fluorescence mode, and 395 fM in the SERS mode (Figure 5b).

It is worth noting that the combined detection of multiple markers of SARS-CoV-2 can further improve the detection sensitivity and accuracy, and some related studies have been published. As a paradigm, Wang et al. provided a rapid and sensitive colorimetric-fluorescent biosensor to detect SARS-CoV-2-specific IgM and IgG by using SiO_2_@Au@QD labels [59]. The biosensor can complete detection within 15 min with 100 times more sensitivity than colloidal gold-based LFIA and only needs 1 μL of serum sample. Likewise, as can be seen from Figure 5c, Wang et al. developed a dual-mode and high-sensitivity fluorescence lateral flow immunoassay system based on Fe_3_O_4_ nanocomposites with a multilayer QD-Shell that can simultaneously detect the S and NP antigens of SARS-CoV-2. The system can achieve rapid direct detection in 10 min, and high-quality detection can be achieved in 35 min via enrichment. The detection limits were 1 and 0.5 pg/mL, respectively, which can meet the requirements of rapid screening and accurate identification [60]. The rapid development of multi-modal biosensors will make the diagnosis more convenient and efficient.

At present, the reported fluorescence sensors can specifically identify and quickly detect the antibodies, proteins, DNA, or RNA of SARS-CoV-2. Particularly, the fastest response time of the biosensor based on fluorescence intensity variations is about 10 min, and the detection limit for the S protein can be as high as 1.6 ng/mL, which can meet the requirements of clinical detection. In addition, the fluorescence biosensor based on imaging technology can rapidly image SARS-CoV-2 in a few seconds, and POC detection can be achieved by combining them with microfluidic techniques. Meanwhile, we found that, compared with traditional fluorescent materials, NIR-responsive fluorescent materials have more advantages in virus detection. Therefore, it is expected to further improve the detection sensitivity and accuracy by developing new near-infrared materials and optimizing the structure and composition of existing materials.

## 4. Colorimetric Biosensors for SARS-CoV-2 Detection

Colorimetry refers to a method that reflects relevant biochemical signals by using the color changes visualized in the detection equipment. It can visually detect biomarkers, such as viruses and proteins, with the help of fluorescent molecules, gold nanoparticles, and other labeled probes. The color change can be observed with the naked eye, even at low concentrations of the target analyte. Simultaneously, colorimetric biosensors have been used in POC for their advantages of direct readout, convenient operation, low instruments requirements, and portability [61,62,63]. In recent years, a series of smart materials that can induce color changes have been developed using noble metals, metal oxides, and carbon materials [64,65]. Among them, metal oxides and carbon materials produce color changes primarily because of the catalyzed reactions of peroxidase substrates or their inherent peroxidase activity. Noble metals, such as gold nanoparticles (AuNPs), platinum nanoparticles (PtNPs), and silver nanoparticles (AgNPs), mainly cause color changes through agglomeration. Notably, gold nanoparticles have been widely studied due to their simple preparation and easy modification. This section will discuss, in detail, the application of colorimetric biosensors based on enzymes or gold nanoparticles in the detection of SARS-CoV-2.

**Figure 6:**
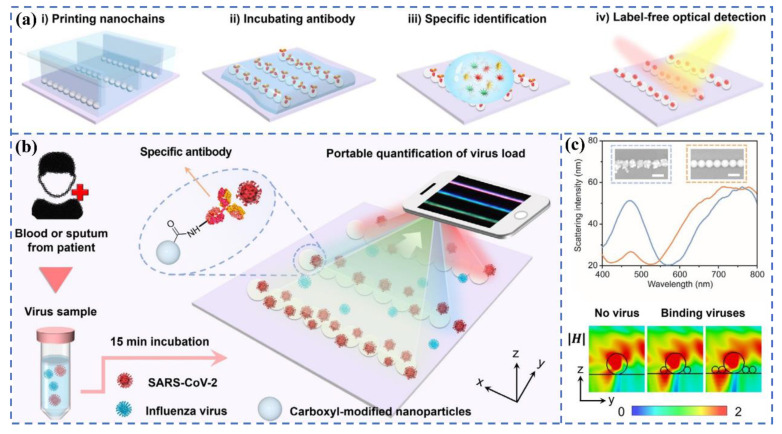
(**a**) Flow chart of the direct colorimetric detection of SARS-CoV-2 in serum or sputum using printed nanochains. (**b**) Quantitative monitoring of virus particles: the color change of nanochains can be observed using a smart phone. (**c**) Scattering spectra and magnetic field distribution before and after viral particle binding. Reprinted with permission from ref. [68]. Copyright 2021, Wiley.

Büyüksünetçi et al. established a practical colorimetric assay for the detection of SARS-CoV-2 based on γ-Fe_2_O_3_ nanoparticles with peroxidase-like activity [66]. The detection principle is that γ-Fe_2_O_3_ particles can oxidize colorless TMB (3,3′,5,5′-tetramethylbenzidine) into blue TMB (Ox). When the SARS-CoV-2 S protein was added to the mixed solution of blue TMB (Ox) and ACE2, the color of the solution gradually became lighter as the concentration of the S protein increased. They speculate that the color change may be caused by the change in the disulfide bond of cysteine in the S protein when TMB (ox), as an oxidoreductase molecule, interacts with TMB (ox) in the RBD region of the S protein and ACE2. Fu et al. reported a novel colorimetric biosensor for the detection of the SARS-CoV-2 S1 protein based on ELISA [67]. The superior peroxidase catalysis of antibody-conjugated Au@Pt NPs enables the sensitive colorimetric detection of the spike (S1) protein of SARS-CoV-2. The dynamic detection range of this sensor is 10–100 ng mL^−1^, and the detection limit is 11 ng mL^−1^, which paves the foundation for the development of high-performance metal nanoparticle-based colorimetric biosensors. Zhang et al. [68] recently prepared one-dimensional nanochain arrays through the template-assisted printing method for the label-free optical visualization detection of SARS-CoV-2 virus particles. The surface of these nanochains has lots of carboxyl groups that can be coupled with specific antibodies, which can quickly capture SARS-CoV-2 virus particles in serum or sputum. According to Figure 6, the color of light scattered by the nanochains will be changed when the virus particles are adsorbed on the nanochains. The quantitative detection of SARS-CoV-2 can be completed within 15 min with the help of smart phones.

In recent years, Au nanoparticles have garnered incredible attention in the field of colorimetric-based biosensing applications owing to their high extinction coefficient, high specificity, and sensitivity. For instance, Rodríguez Díaz et al. proposed a fast, reliable, and low-cost colorimetric sensor based on AuNPs for the selective detection of the RdRp, E, and S proteins of SARS-CoV-2. The sensor can complete detection within 15 min and maintain high activity even after being placed for several months. Therefore, it can be used for the early diagnosis of SARS-CoV-2 [69].

The most significant advantage of the colorimetric biosensor based on colorimetry is that the presence of target substances in the system can be judged by the naked eye, and the detection limit of the new colorimetric sensor developed can reach 1 PFU/µL, which is very suitable for home detection application scenarios. However, colorimetry can only perform qualitative analysis and has poor stability between different batches of products. Therefore, in the future, colorimetry needs to be combined with other technologies (such as electrochemistry, SERS, etc.) to further improve the sensitivity and accuracy of the detection system. Table 1 summarizes some significant parameters of fluorescence biosensors, including the matrix, target analyte, detection time, limit of detection, and detection sensitivity.

## 5. SERS Biosensors

Raman spectroscopy is a type of spectroscopy that can characterize the vibrations of chemical bonds of molecules. However, the intensity of scattered light collected by Raman spectroscopy is lower than that of the incident light, resulting in a weaker Raman signal [70]. Therefore, SERS was introduced to address the inherent weak signal weakness of Raman spectroscopy. The enhancement mechanism of SERS consists mainly of electromagnetic enhancement and chemical enhancement, which are caused by the surface plasmon resonance of the substrate and the charge transfer between the substrate and the probe molecules, respectively [71,72]. Surface-enhanced Raman detection technology has a high sensitivity, and the detection ability of some SERS-active substrates can even reach the single molecule, which has been widely used in cancer detection, virus detection, biological imaging, and other medical fields [73,74,75,76]. Recently, SERS has gained much attention in the rapid detection of SARS-CoV-2 and the infectivity judgment of SARS-CoV-2 fields due to its high sensitivity, accuracy, rapidity, and low cost.

SERS technology is divided into two categories, namely, labeled SERS technology and label-free SERS technology. Labeled SERS technology involves labeling the reporter molecules with a higher Raman scattering cross-section on the substrate. The Raman signal of the reporter molecule is proportional to the concentration of the analyte by reasonably designing the SERS detection system, and then the concentration information of the target molecule is finally obtained. The labeled method is suitable for the molecular vibrations of the target molecules without or with weak Raman activity, so the substance to be tested can be indirectly detected by a labeled reporter molecule with strong Raman activity. However, the design of the labeled SERS technology is relatively complicated, and the molecular structure of the substance to be tested cannot be analyzed because the Raman signal of the substance to be tested is not directly collected, which leads to the loss of the advantage of Raman as fingerprint spectra. In addition, the substrate design of the labeled method is more complicated, and the stability of the substrate performance is difficult to guarantee.

The label-free SERS technique directly collects the Raman spectra of the substance to be tested, and the molecular structure of the substance to be tested can be analyzed by analyzing the corresponding Raman vibrational spectra. Especially, by distinguishing different viruses and identifying virus variants, not only can different viruses be distinguished from the perspective of spectral vibration, but the actual mutation properties of viral nucleic acids and proteins can be further analyzed and verified. However, for the label-free SERS detection of biological macromolecules, the weak spectral signal strength of biological macromolecules and the poor spectral reproducibility caused by different adsorption directions are major challenges [77,78,79].

### SERS Biosensors for SARS-CoV-2 Detection

**Figure 7:**
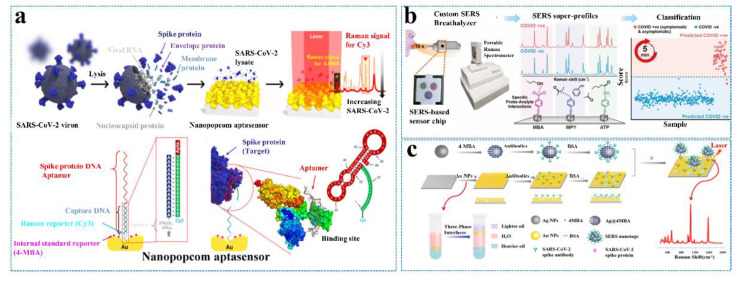
(**a**) Schematic illustration of the quantitative evaluation of SARS-CoV-2 using the SERS-based biosensor. Reprinted with permission from ref. [80]. Copyright 2021, American Chemical Society. (**b**) Overview of the SERS-based biosensor to identify COVID-19 patients using their breath volatile organic compounds (BVOCs). Reprinted with permission from ref. [81]. Copyright 2022, American Chemical Society. (**c**) Schematic illustration of the SERS-based immunoassay. Reprinted with permission from ref. [85]. Copyright 2021, Elsevier.

With the outbreak of COVID-19, China, the United States, South Korea, and Singapore have pioneered the development of SERS detection technology for SARS-CoV-2. Yang’s group [70] reported the Raman characteristic spectra and determination criteria of SARS-CoV-2. This is the first report of the excellent SERS activity of Nb_2_C material and the accurate identification of Raman peaks of the SARS-CoV-2 S protein. It is of great significance for the real-time monitoring and early warning of SARS-CoV-2. The team from Chung-Ang University in South Korea reported that the aptamer-modified SERS sensor was applied to detect SARS-CoV-2 with an LOD of 10 PFU/mL for 15 min (Figure 7a) [80]. Recently, a research group from the Nanyang Technological University designed a breath analysis model based on SERS which can complete the screening of SARS-CoV-2 within 5 min (Figure 7b) [81]. A novel SARS-CoV-2 biosensor based on SERS has been developed by using large-area nanoimprint lithography at Johns Hopkins University, an authority on SARS-CoV-2 research in the United States. The biosensor combines machine learning technology to simultaneously improve detection accuracy and detection speed, which is especially suitable for large-scale group detection. However, its sensitivity can only be improved to a level of 10^3^ copies/mL after improvement [82].

The Labeled SERS technology cannot directly obtain the spectral information of the substance to be tested, but this method has good quantitative characteristics, that is, the Raman intensity of the SERS reporter has a strong linear relationship with the concentration of the analyte [83,84]. Because of its high sensitivity and rapidity, the labeled SERS detection platform has achieved wide applicability in the rapid detection of SARS-CoV-2.

Liu et al. [85] prepared Au nanoparticle-assembled bilayer membranes by self-assembly at the oil/water/oil-liquid interface. The surface of Au foil modified with the SARS-CoV-2 antibody can be employed as an immune substrate to detect the SARS-CoV-2 antigen (Figure 7c). The group also designed a labeled immuno-SERS detection platform, which is a “sandwich” structure consisting of an immuno-SERS substrate, an analyte, and the SERS tag of antibody-modified Ag nanoparticles. The SERS-based immune platform is not disturbed by impurities in the environment, and the LOD of the SARS-CoV-2 S protein in untreated saliva can reach 6.07 fg/mL. The assay platform has excellent specificity and reproducibility. Ray et al. [86] prepared antibody-modified Au nanoparticles to detect the SARS-CoV-2 S protein and pseudo virus. They used gold clusters of different sizes to present different plasmon resonance peaks, thus exhibiting different color features. They were able to quickly identify the presence of SARS-CoV-2 with the naked eye using colorimetry, which is highly sensitive, with an LOD of 1 ng/mL for the S protein and of 1000 vp/mL for the pseudo virus. The research found that it was difficult to combine with ACE2 for SARS-CoV-2 when the S protein was combined with the antibody-modified Au nanoparticles. It can be seen that the antibody-modified Au nanoparticles can be used as a nano-vaccine, which is expected to realize a strategy integrating detection and treatment.

The labeled method mainly detects SARS-CoV-2 indirectly by detecting the signal of the reporter molecule in the SERS tag, so the selection of the reporter molecule and the construction of the SERS-active substrate are very critical. Usually, nano-scale Au, Ag, and their composite with strong electromagnetic field enhancement are utilized as the substrate, and 4-mercaptobenzoic acid (4-MBA), 4-aminothiophenol, rhodamine 6G (R6G), etc. containing -SH/-NH_2_ groups are applied as reporter molecules. Strong binding Ag/Au-S/N bonds caused by strong electrostatic interactions are employed to construct SERS tags.

**Figure 8:**
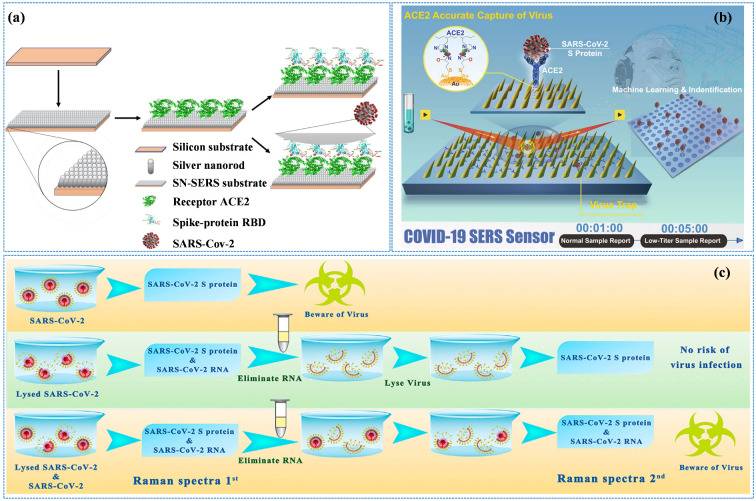
(**a**) State-of-the-art diagram of SERS sensing for interrogating SARS-CoV-2. Reprinted with permission from ref. [88]. Copyright 2021, Elsevier. (**b**) Schematic diagram of the operation procedure of COVID-19 SERS sensors. Reprinted with permission from ref. [89]. (**c**) The schematic of the two-step SERS detection based on SnS_2_ microspheres for diagnosing the infectiousness of SARS-CoV-2. Reprinted with permission from ref. [90]. Copyright 2022, Elsevier.

The label-free SERS technology can not only rapidly screen and diagnose SARS-CoV-2 carriers but also further analyze the spectral information of the SARS-CoV-2 nucleic acid, antigen, antibody, and pathogen, including distinguishing virus types, identifying SARS-CoV-2 variants, judging the virus infectivity, etc. Daoudi et al. [87] prepared silver nanoparticle/silicon nanowire (AgNPs/SiNWs) complexes and achieved an LOD of 10^−12^ mol/L for RBD in the SARS-CoV-2 S protein by optimizing the length of SiNWs and regulating the AgNPs immersion time. In this work, the SERS spectra of RBD were poorly reproducible, mainly due to the different adsorption sites of biomacromolecules on the surface of the SERS substrate. To obtain better spectral repeatability, the researchers targeted the capture of the analyte by modifying ACE2 or other specific antibodies on the SERS substrate. In addition, the specifically modified ACE2 or antibody will also endow SERS substrate specificity, thus effectively avoiding the interference of impurities in the physiological environment. Wang et al. [88] reported a kind of SERS biosensor modified with ACE2 to detect SARS-CoV-2 in medical sewage, and its accuracy reached 93.33% (Figure 8a).

The Shanghai Institute of Ceramics, Chinese Academy of Sciences has achieved a series of pioneering research results in the field of the precise capture and detection of SARS-CoV-2 by SERS technology. The developed sensor has an ACE2-functionalized gold nano “forest” structure which can selectively capture SARS-CoV-2, with the detection sensitivity reaching the level of a single virus (Figure 8b) [89,90,91]. The sensor has a 10^6^-fold improvement ability to enrich viruses in water due to the special nano “forest” structure and the high affinity of ACE2 for the S protein of SARS-CoV-2. In addition, virus diagnostic signal criteria and methods are established through machine learning methods. Its optimal LOD for SARS-CoV-2 reaches 80 copies/mL, and the detection time is only 5 min, which is of great significance for the SARS-CoV-2 clinical testing. The research group proposed a two-step SERS detection method based on an ultrasensitive SnS_2_ microsphere substrate, as shown in Figure 8c, and established the SERS signal identification criteria for the SARS-CoV-2 S protein and RNA, which were used to identify SARS-CoV-2 infectiousness in the environment. It solved the inability of PCR technology to diagnose the infectiousness of SARS-CoV-2 in the environment. The biosensor is of great significance to avoid misjudgment in the situation of SARS-CoV-2 raging [90].

SERS biosensors are considered one of the potential methods for POC diagnosis due to the merits of the ultra-fast detection time, high sensitivity, and accuracy. As shown in Table 2, the reported developed SERS sensor can even complete the detection within 1 min, and the detection limit can reach 10^−12^ mol/L. However, some of the vibrational Raman signals are weaker than those of dye molecules because of the small Raman scattering cross-section of biomolecules. Additionally, affected by impurities in a different physiological environment, the adsorption direction of bio-macromolecules on the surface of the SERS substrate is different. Therefore, the label-free SERS biosensor mainly detects the S protein of SARS-CoV-2 and the complete SARS-CoV-2. As we all know, the SERS substrate is very important in obtaining SERS sensors with excellent performance. Therefore, the detection of multiple virus markers and the further improvement of the detection sensitivity and accuracy are expected to be realized by optimizing the structure and morphology of the substrate.

## 6. SPR-Based Biosensors

Propagating SPR is a part of the physical optical phenomenon, which is caused by the resonance between the polarized incident light and plasma wave induced by electron oscillation on the metal surface [92]. When polarized light strikes the interface between the prism and the metal at the angle of incidence (θ1), a part of the light penetrates into the metal film and forms an evanescent wave, which induces electrons in the metal to generate surface plasmon polariton [93]. The incident light energy is resonantly transferred when the frequencies of the two waves are the same, resulting in the phenomenon of surface plasmon resonance. The incidence angle occurring at the resonance phenomenon is called the resonance angle, which varies with the surface refractive index [94]. The SPR sensors based on the above principle can record the shift of the resonance angle according to the change in the surface refractive index, when the analyte is adsorbed or dissociated on the metal surface, consequently realizing the real-time monitoring of surface intermolecular interactions and the quantitative detection of targets [95,96]. SPR-based sensors can be divided into four categories according to the nature of the detected reflected light: intensity-modulated SPR biosensor [97], angle-modulated SPR biosensor [98], wavelength-modulated SPR biosensor [99], and phase-modulated SPR biosensor [100]. According to the different coupling structures, SPR can be divided into prism-based coupling structures [101], optical wavelength-based coupling structures [102], grating-based coupling structures [103], and optical fiber-based coupling structures [104].

Compared with traditional detection technology, SPR detection technology has outstanding advantages [105,106,107]. (1) Label-free feature. The SPR technology can recognize the target substance by the specific binding between the ligand and the receptor without the need to label the specific ligand, thus ensuring the in situ of the sample to the utmost extent. (2) Good dynamic performance. The SPR sensor can monitor the dynamic process of the reaction in real time and continuously, and it is suitable for turbid, opaque, or colored liquids. (3) Good stability. The SPR sensor has a high stability for the detection of samples with different refractive indices and is suitable for the detection needs in a variety of complex environments. (4) Wide range of applications. SPR sensors can be used for the qualitative and quantitative detection of gases, liquids, and bio-macromolecules and the real-time dynamic monitoring of samples to be tested.

### 6.1. SPR Biosensors for SARS-CoV-2 Detection

**Figure 9:**
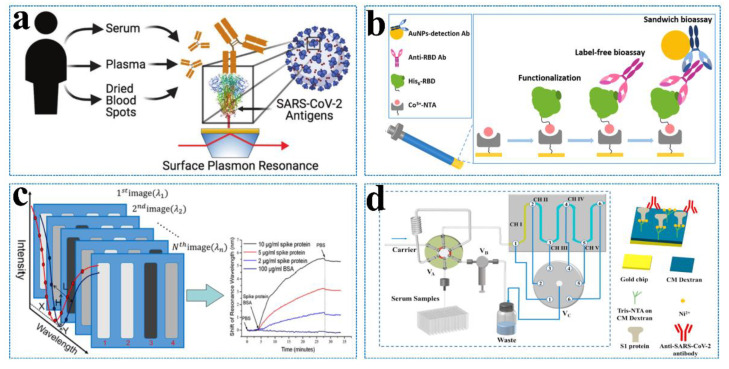
(**a**) A series of SPR sensors modified with different SARS-CoV-2 antigens (nucleocapsid, RBD, or spike) is applied to detect human antibodies from different blood products (serum, plasma, or dried blood spots) collected from COVID-positive or -negative individuals. Reprinted with permission from ref. [108]. (**b**) Label-free FO-SPR serological bioassays with sandwich formats for the detection of anti-SARS-CoV-2 RBD antibodies. Reprinted with permission from ref. [110]. Copyright 2022, American Chemical Society. (**c**) Schematic diagram of the fast wavelength interrogation SPR imaging biosensors. Reprinted with permission from ref. [111]. Copyright 2021, American Chemical Society. (**d**) Schematic of the five-channel SPR system with an autosampler for detecting the anti-SARS-CoV-2 antibody in serum samples (left). Captures of the anti-SARS-CoV-2 antibody by the His-tagged S1 protein pre-immobilized onto the CM-dextran-based tris-NTA sensor (right). Reprinted with permission from ref. [112]. Copyright 2022, Elsevier.

A variety of detection platforms have been proposed for the detection of SARS-CoV-2 and related analytes, among which SPR sensing is very suitable for the quantitative analysis of the novel coronavirus antigen, antibody, and RNA and the monitoring of antigen–antigen and antigen–antibody interactions. Djaileb et al. (Figure 9a) [108] designed SPR sensors coated with polypeptide and SARS-CoV-2 recombinant S protein expressed by different cell lines to detect the SARS-CoV-2 IgG antibody in clinical samples. The N protein in different cell lines has little effect on the detection of antibodies, while the S protein in the CHO cell line will lead to a better performance. The above biological analysis adopted a portable SPR device, which can complete the detection of four samples within 30 min. Optical fiber SPR biosensors are also widely used in SARS-CoV-2 detection.

The fiber-optic SPR sensor can amplify the wavelength modulation operation for optical excitation. It is suitable for remote sensing, on-site monitoring, and in vivo measurements due to the smaller sample, low cost, miniaturization, and simpler structure. Cennamo et al. [109] constructed optical fiber biosensors with a limit of detection of 37 nM for the SARS-CoV-2 spike protein. Although the sensor will not be affected by interferents (BSA, AH1N1 hemagglutinin protein, and MERS spike protein), the detection sensitivity needs to be further improved. Qu et al. (Figure 9b) [110] constructed a label-free fiber SPR-based sensor to directly detect antibodies in undiluted whole blood, which can achieve the entire detection within 30 min. Optical fiber SPR sensing has demonstrated the capability to work at the nanoscale and has demonstrated versatility in terms of design, power consumption, materials, and sensing performance. However, conventional-shaped fiber SPR sensors are not suitable for viral detection at a low viral load. Specific-shaped fiber-SPR sensors are more attractive for satisfying the detection requirements.

SPR sensors can also achieve high throughput to meet the needs of practical detection. Wang et al. [111] (Figure 9c) constructed a spectra-based SPR sensor device to achieve the high-throughput detection of the SARS-CoV-2 S protein, proving its application possibility in screening COVID-19 with a high accuracy. Jiang et al. (Figure 9d) [112] combined microfluidic technology and SPR sensing to develop a high-throughput antibody detection device. The device has five channels that can be detected at the same time. Each sensor channel can be reused 20 times and has excellent reproducibility (RSD ≤ 2.1%) and sensitivity (57 ng/mL) for the detection of SARS-CoV-2 antibodies. Chen et al. [113] proposed the combination of SPR sensing technology and Clustered Regularly Interspaced Short Palindromic Repeat (CRISPR) technology as MOPCS (Methodologies of Photonic CRISPR Sensing) for the first time. MOPCS can be used for the high-sensitivity detection (15 fm) of SARS-CoV-2 RNA, which can be from sample input to results within 38 min without amplification. In addition, MOPCS can realize the SARS-CoV-2 variants B.1.617.2 (Delta), B.1.1.529 (Omicron), and BA.1 (a subtype of Omicron).

In the prevention and control of COVID-19, it is also crucial to develop tools that can quickly and accurately judge the infectivity and vaccine efficiency. SPR sensing technology can monitor molecular interactions and reflect information about their dynamic. Susanne N. Walker et al. [114] utilized SPR sensing technology to quantify the antibodies in serum that bind to the COVID-19 S protein and inhibit ACE2. The Center for Biologics Evaluation and Research (CBER) [115] used S1 + S2 ectodomain, S1 domain, RBD, and S2 domain as antigens, respectively, and SPR sensors were applied to quantitatively explore the antibodies induced by the above antigens. The results show that, compared with other S proteins, RBD can elicit a five-fold antibody titer when it acts as an immunogen. The institution also used SPR sensors to evaluate the binding ability and affinity of the S proteins of WA1, BA.1, and BA.2 to antibodies [116]. SPR sensing technology can realize the characteristics of immune kinetics and can screen drugs that are compatible with ACE2 or the S protein so as to achieve the purpose of treatment [117]. Singh et al. [118] utilized SPR to evaluate the interaction between components of Momordica charantia (erythrodiol) and the S protein. The results showed that erythrodiol had a strong binding affinity with the S2 protein region of SARS-CoV-2 (Kd = 1.15 µm). The components in Momordica charantia have the potential to be used as reagents to treat COVID-19 infection due to their binding with the S protein. SPR sensors were used to characterize the binding affinity between the component of traditional Chinese medicine, epigallocatechin-3-gallate (EGCG), and the protease of SARS-CoV-2, 3CLpro, by Du et al. [119]. Wang et al. [120] designed and synthesized phenanthridine derivatives that specifically bind to the N protein. SPR sensors were used to screen these derivatives, and the optimal binding affinity could reach 2.18 µM. SPR sensing can be used to screen substances related to S protein/N protein/protease/ACE2 in order to inhibit SARS-CoV-2 infection and achieve the purpose of treatment, which is of great significance for the prevention and control of COVID-19.

### 6.2. LSPR Biosensors for SARS-CoV-2 Detection

In order to manufacture chips for SPR sensors, complex instruments such as sputtering coaters or vacuum evaporators are usually required to coat the surface of optical prism-/fiber-substrates with noble metal films to excite the SPR. In recent years, Surface Plasmon Resonance (LSPR)-based biosensors have also attracted more and more attention. As an SPR phenomenon, they exist in metal nanoparticles (MNPs) rather than bulk metals. LSPR is an optical phenomenon produced by the interactions between incident light and MNPs or metal conductive electrons smaller than the incident wavelength. Its resonance frequency strongly depends on the material, size, geometry, and detection environment of MNPs and on the spacing of MNPs. LSPR sensors have advantages in the following respects: (1) In terms of the sensing principle, the sensing data of LSPR are detected according to the reflectance and absorption spectrum, which can be realized only with ordinary laboratory equipment. (2) In terms of manufacturing devices, LSPR sensors have a more flexible design and a lower cost because LSPR can be excited when incident light directly interacts with MNPs without prism or other optical elements. (3) In terms of the detection mode, nanoparticles have a highly concentrated LSPR sensing area, which eliminates the need for the polymer substrate to enhance the signal. Moreover, since LSPR can be generated in single MNPs, LSPR sensors can even be designed based on a single nanoparticle. In terms of detection performance, LSPR sensors show high sensitivity, which makes them particularly attractive in applications. LSPR-based biosensors have also been widely applied in the detection of SARS-CoV-2.

**Figure 10:**
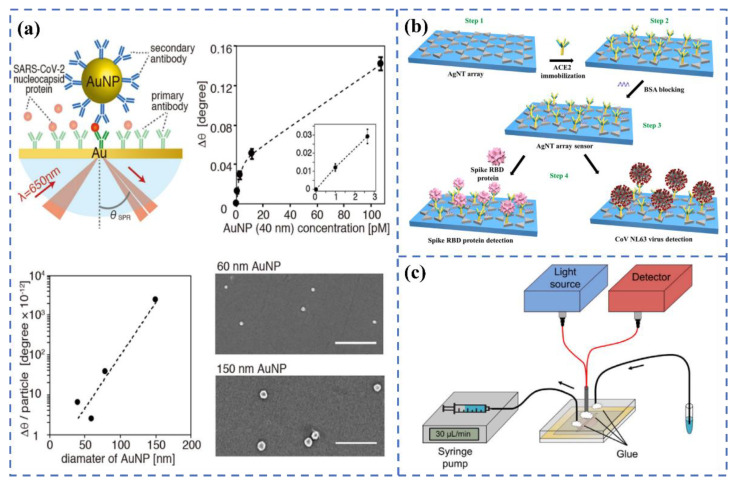
(**a**) Principle of the LSPR biosensor for detecting SARS-CoV-2 nucleocapsid protein using gold nanoparticles. Reprinted with permission from ref. [121]. (**b**) Schematic diagram strategy for spike RBD protein or virus detection. Reprinted with permission from ref. [122]. Copyright 2022, Elsevier. (**c**) The diagram of the opto-microfluidic SPR system. Reprinted with permission from ref. [123]. Copyright 2020, Elsevier.

Yano et al. [121] (Figure 10a) constructed the sandwich structure of Au substrate-SARS-CoV-2 N protein-antibody-modified Au nanoparticles, and the detection sensitivity of the N protein reached the fmol/L level through LSPR sensing technology. The group attributed the excellent sensitivity of the system to the coupling effect of the surface plasmon resonance between Au nanoparticles and Au substrates and the utilization of large-sized Au particles (about 150 nm). Through experiments and electromagnetic field simulation, they believed that ~150 nm Au particles were more conducive to the detection of bio-macromolecules than tens of nm of Au nanoparticles. Yang et al. [122] (Figure 10b) constructed an LSPR sensor with ACE2-functionalized Ag nanotriangles to achieve the high-sensitivity detection (0.83 pM) of RBD within 20 min. Funari et al. [123] (Figure 10c) constructed a microfluidic-based LSPR sensing device for the quantitative detection of antibodies, which is specific to the S protein of SARS-CoV-2. The device has excellent sensitivity, with an LOD of 0.08 pg/mL, and it can complete the detection within 30 min. Yokoyama et al. [124] studied the adsorption process of the Omicron S protein by monitoring the change in the SPR peak shift of gold sol with a particle size of 10–100 nm. The results have shown that when the size of the gold nanoparticles was larger than 30 nm, the fluctuation of the peak position would occur.

**Figure 11:**
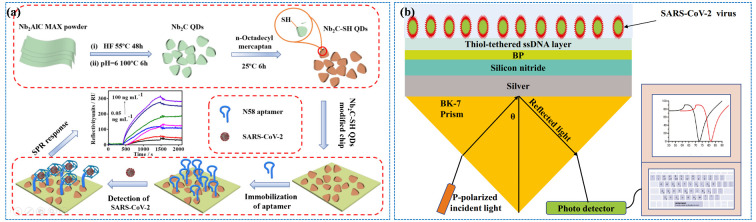
(**a**) Schematic of the synthesis of Nb_2_C-SH QDs (top) and the construction of the Nb_2_C-SH QD-based SPR aptasensor for detecting the SARS-CoV-2 N gene (bottom). Reprinted with permission from ref. [125]. Copyright 2021, Springer. (**b**) The configuration of an SPR sensor based on prism-Ag-Si_3_N_4_-BP-ssDNA. Reprinted with permission from ref. [126]. Copyright 2022, Springer.

Whether SPR or LSPR, most of them utilize noble metals as sensing elements in the field of SARS-CoV-2 detection. Some low-cost semiconductor materials have also been developed for the detection of SARS-CoV-2. Rong Chen et al. [125] used niobium carbide mxene quantum dots (Nb_2_C-SH QDs) to construct an SPR aptasensor for detecting the N gene of SARS-CoV-2, as shown in Figure 11a. An Nb_2_C-SH QDs-based SPR aptasensor has excellent sensitivity to the N gene, and the LOD is 4.9 pg/mL. Kumar et al. [126] constructed an SPR biosensor based on hybrid materials (Si_3_N_4_-BP-Ag), which showed an excellent sensitivity (154°/RIU) compared with other configurations (Figure 11b). Table 3 summarize the representative SPR biosensors for SARS-CoV-2 virus detection.

**Figure 12:**
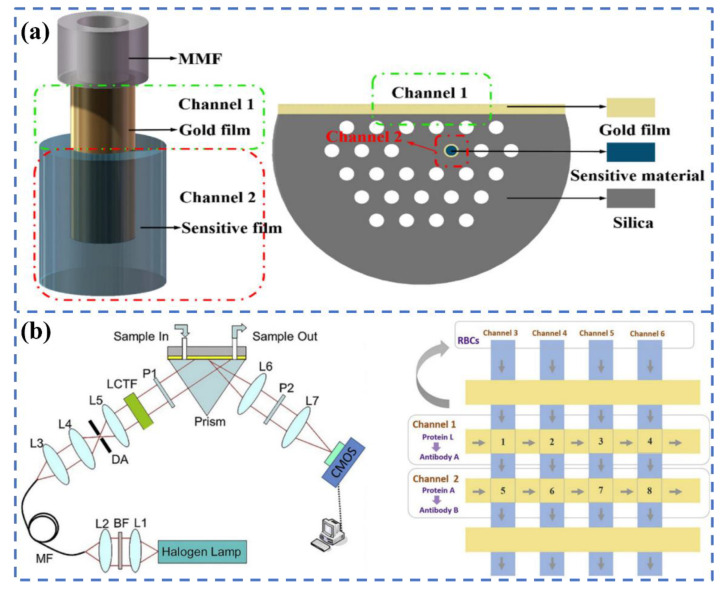
(**a**) Schematic diagram of the multi-channel SPR sensor. Reprinted with permission from ref. [129]. Copyright 2022, Wiley. (**b**) Diagram of the SPR imaging system for the detection of blood typing. Reprinted with permission from ref. [130]. Copyright 2020, Elsevier.

SPR sensing can not only be used for the detection of SARS-CoV-2 but can also monitor the binding dynamics of protein–protein and protein–antibodies, which plays a guiding role in the mechanism and treatment of SARS-CoV-2 infection. Although SPR technology has the advantages of being label-free and having a high sensitivity and fast response, it is still necessary to further improve it in practical application. Due to the complex matrix of the actual system, the interferents such as proteins, lipids, polysaccharides, and so on are easily adsorbed on the sensing surface, resulting in obvious signal interference, affecting the specific adsorption and recognition of target molecules and seriously reducing the sensitivity of the sensors and the reliability of the detection results [127,128]. In addition, due to the wide variety of analytes in blood, food, and other samples, some samples are scarce and expensive in the specific detection. Common and single analyte detection methods have a low efficiency and a large sample consumption, which increases the detection cost to a certain extent and severely limits the application of traditional SPR sensors. Strengthening the elimination of biological pollution in the sensing area and the construction of the anti-pollution surface, as well as developing efficient and high-throughput analytical methods, are of great significance for the application of SPR sensors. Figure 12 shows two methods to improve SPR detection throughput, namely, using multi-channel detection (a) and SPR imaging (b) [129,130]. In addition, semiconductor materials can optimize their SPR performance through morphology regulation, doping modification, and hybrids with precious metals so as to develop practical applications based on semiconductor SPR sensing.

## 7. Conclusions and Prospects

Since the outbreak of the pandemic in 2019, COVID-19 has spread globally and has had a dramatic impact on the world. Furthermore, the coronavirus is constantly mutating, spreading faster and more insidiously, and presenting problems of globalization and time persistence. The development of rapid virus detection and diagnosis technology is of great significance to preventing virus transmission. The traditional RT-PCR detection method is unable to meet the needs of fast, high-throughput, and on-site detection because of its time-consuming and strict requirements regarding personnel and equipment. There is a growing importance of developing rapid, simple, high-throughput, and intelligent detection methods, especially in some developing countries and regions that are more vulnerable to the epidemic and which lack medical resources [131,132,133]. Optical biosensors provide an ideal selection for the detection of SARS-CoV-2. In this review, we discuss the recent research progress of optical sensors in the field of SARS-CoV-2 detection, and some key parameters of representative optical sensors are summarized. As can be seen from Table 1, the average detection time of fluorescent sensors is about 20 min, and the detection limit can reach the level of nM. It is worth pointing out that, compared with single-modal biosensors such as fluorescence resonance energy transfer biosensors, the sensitivity of multi-modal biosensors is significantly increased, which can meet the needs of different scenarios, but the structural design of the matrix is relatively complex. As a traditional optical sensor, the colorimetric biosensors exhibit high sensitivity, but the bottleneck of only using qualitative analysis limits their application range. Additionally, according to Table 2, it is not difficult to see that the SERS biosensors show an ultra-fast response and excellent sensitivity. However, the poor stability of the SERS substrate makes it difficult to guarantee the reproducibility of various batches of biosensors. Although SPR biosensors possess ultra-high sensitivity, they are time-consuming and unable to meet the requirements of POC detection (Table 3).

To speed up the development of optical sensors, efforts can be made from the following perspectives for the development of new optical biosensors:

(1) More green synthesis strategies should be introduced to prepare low-toxicity, non-polluting optical materials to satisfy the requirements of sustainability.
(2) The advantages of various materials should be fully used to study multifunctional optical materials and improve the sensitivity and accuracy of diagnostics to meet the needs of different detection scenarios.
(3) The mutations of the virus seriously interfere with the accuracy of detection. Thus, improving the recognition and detection ability of multiple pathogens and virus variants can save a lot of manpower and material resources under normal management.
(4) For different application scenarios such as hospitals, customs, communities, and even families, the development of portable, economical, and miniaturized instruments to achieve rapid on-site detection will greatly help enhance the timeliness of detection and reduce the risk of the spread of SARS-CoV-2. It is reasonable to believe that optical biosensors will play an important role in the future detection of SARS-CoV-2 and even unknown viruses through the aforementioned efforts.

## Figures and Tables

**Table 1:** Matrix, target analyte, detection time, limit of detection, and detection sensitivity of the latest fluorescence biosensors.

Matrix	Target Analyte	Detection Time	Limit of Detection	Detection Sensitivity	Ref
CdTe/ZnS QDs	DNA/RNA	25 min	0.823 nM	-	[38]
Cy3 organic dye	DNA	20 min	0.09945 μM	-	[40]
NaYF_4_: Yb,Er@SiO_2_ nanoparticles	S/N proteins	10 min	1.6 ng/mL(S) 2.2 ng/mL (N)	-	[47]
Eu fluorescent microsphere	IgM and IgG	10 min	-	98.72% (IgG) 98.68% (IgM)	[48]
Ln^3+^-polysterene nanoparticle	IgG	10 min	-	94.74%	[49]
NIR-Ⅱnanoparticles	N proteins	15 min	0.01 ng/mL	-	[50]
Single-walled carbon nanotubes	S protein	imaged in seconds	12.6 nM	-	[52]
NaYF_4_: Yb, Er/graphene oxide	RNA	30 min	5 fM	-	[53]
SiO_2_@Au/QD	S protein	30 min	1 ng/mL-colorimetric 33 pg/mL-fluorescence	-	[56]
AgMBA@Au	IgG	15 min	10^−7^ mg/mL-colorimetric	-	[57]
Au nanoparticles	RNA	40 min	160 fM-colorimetric 259 fM-fluorescence 395 fM-SERS	-	[58]
SiO_2_@Au@QD	IgM and IgG	15 min	-	100% (IgG) 100% (IgM)	[59]
γ-Fe_2_O_3_ nanoparticle	S protein	20 min	4.98 ng/mL	-	[66]
Au@Pt nanoparticle	S protein	20 min	11 ng/mL	-	[67]
Polystyrene nanoparticles	SARS-CoV-2 virus particles	15 min	1 PFU/µL	-	[68]
Au nanoparticles	E/S protein	2.5 h	10^3–^10^4^ viral RNA copies/µL	-	[69]

**Table 2:** Some key parameters of the reported SERS biosensors for SARS-CoV-2 detection.

Matrix	Target Analyte	Detection Time	Limit of Detection	Detection Sensitivity	Ref
Nb_2_C	S protein	15 min	10 PFU/mL	-	[80]
Ag nanocubes	breath volatile organic gas	5 min	-	96.2%	[81]
Ag	fusion protein	25 min	10^3^ copies/mL	-	[82]
Au membranes	S protein	-	6.07 fg/mL	-	[83]
Au nanoparticles	S protein	5 min	1 ng/mL	-	[86]
Ag nanoparticles/siliconnanowires	S protein	<1 min	10^−12^ mol/L	-	[87]
SnS_2_ microsphere	S protein	5 min	80 copies/mL	-	[90]

**Table 3:** Characteristic parameters of some representative SPR biosensors for SARS-CoV-2 virus detection.

Matrix	Target Analyte	Detection Time	Limit of Detection	Detection Sensitivity	Ref
Au	S protein	30 min	37 nM	-	[109]
Au	IgG	30 min	-	-	[110]
Au chip	antibodies	30 min	57 ng/mL	-	[112]
Au	RNA	38 min	15 fM	-	[113]
Au nanospike	S protein	30 min	0.08 pg/mL	-	[123]
Nb_2_C-SH QDs	N gene	>15 min	4.9 pg/mL	-	[125]

## Data Availability

Not applicable.
